# Increase in NF-κB-sensitive miRNA-146a and miRNA-155 in multiple sclerosis (MS) and pro-inflammatory neurodegeneration

**DOI:** 10.3389/fnmol.2015.00005

**Published:** 2015-03-02

**Authors:** Deidre J. Devier, Jesus F. Lovera, Walter J. Lukiw

**Affiliations:** ^1^Department of Cell Biology and Anatomy, Louisiana State University Health Sciences CenterNew Orleans, LA, USA; ^2^Department of Neurology, Louisiana State University Health Sciences CenterNew Orleans, LA, USA; ^3^Neuroscience Center of Excellence, Louisiana State University Health Sciences CenterNew Orleans, LA, USA; ^4^Department of Ophthalmology, Louisiana State University Health Sciences CenterNew Orleans, LA, USA

**Keywords:** autoimmunity, degeneration, multiple sclerosis, microRNA (miRNA), miRNA-146a, miRNA-155, inflammation, NF-kB (p50/p65)

## Overview

Multiple sclerosis (MS) is a complex, debilitating, immunopathologic disease of the human central nervous system (CNS) characterized by chronic systemic inflammation, alterations in innate-immune signaling, progressive demyelination and axonal loss. Currently there is no effective treatment or cure for MS. Recent data indicate that common molecular-genetic mechanisms involving a select group of NF-kB-sensitive microRNAs are shared by most MS patients, and their mechanism of pathogenic action is becoming increasingly understood. This brief “Opinion” paper will highlight some recently clarified roles for two NF-kB-regulated, pro-inflammatory microRNAs, miRNA-146a and miRNA-155, in the MS disease process. We will also advance an opinion on how anti-NF-kB, anti-miRNA and/or related therapeutic strategies may be beneficial in the clinical management of MS and other progressive CNS diseases exhibiting inflammatory neurodegeneration.

## Multiple sclerosis—incidence, symptoms, and autoimmunity

To understand the contribution of miRNA-146a and miRNA-155 to the etiopathogenesis of MS we will briefly highlight some salient features of this autoimmune disease. MS is globally the most common immunopathologic disorder affecting the human CNS: (i) with about 2.5 million affected worldwide (approximately 30 cases/100,000 globally; http://www.nationalmssociety.org/); (ii) with a variable incidence of frequency in different regions of the world (Melcon et al., 2014); (iii) with a variable incidence of occurrence amongst different human populations (Høglund and Maghazachi, 2014); (iv) with significant heterogeneity in the clinical phenotype (Sturm et al., 2014; http://www.nationalmssociety.org/); and (v) with low concordance rates in monozygotic twins, suggesting the involvement of complex heritable, epigenetic, microbial and/or environmental factors (Küçükali et al., 2014; Ma et al., 2014; Melcon et al., 2014). MS is generally characterized by abnormal responses of the immune system directed against glial-derived CNS myelin which normally sheaths, insulates and protects axons and nerve bundles (Küçükali et al., 2014; Melcon et al., 2014). MS exhibits (i) “*sclerotic*” or *lipoprotein-enriched scar tissue nodules* associated with demyelinated axons where normal electrical activities become progressively disorganized (Baranzini, 2014; Küçükali et al., 2014); and (ii) a wide variety of clinical symptoms that include muscle weakness, spasticity, loss of balance, sensory deficit and fatigue, dizziness, and vertigo, bowel, bladder and visual problems, depression and cognitive and psychiatric changes; approximately 90% of MS individuals become ultimately disabled (Baranzini, 2014; Guo et al., 2014; Harris and Sadiq, 2014). Consistent observations at the pathogenic and molecular-genetic level indicate five main *highly interactive* characteristics of MS: (i) a progressive demyelination whose extent correlates to MS severity; (ii) axonal swelling and macrophage activation; (iii) a T-cell mediated inflammatory response that subsequently triggers immune cells to release pro-inflammatory cytokines such as IL-1β; (iv) permeability changes in the blood–brain barrier (Kamphuis et al., 2015); and (v) increases in pro-inflammatory microRNA and related pathogenic biomarkers (Haghikia et al., 2012; Meinl and Meister, 2012; Danborg et al., 2014; Harris and Sadiq, 2014; Küçükali et al., 2014; Ma et al., 2014; Sturm et al., 2014; Kamphuis et al., 2015; see below). Specific gene mutations linked to MS include a cluster at human chromosome 6, part of the so-called “autoimmunome” network, which serves as the major histocompatibility complex (MHC) locus; interestingly this genetic locus is also implicated in the autoimmune disease type 1 diabetes and systemic lupus erythematosis (Baranzini, 2014; Gourraud et al., 2014; Sturm et al., 2014). Epidemiological evidence collectively indicates that MS is an immunopathologic disorder initiated by exogenous factors including microbes, possibly of viral origin, vaccines or unknown environmental factors in susceptible individuals genetically predisposed for MS (Gilden, 2005; Küçükali et al., 2014; Ma et al., 2014; Sturm et al., 2014). Indeed, heterogeneity in the MS clinical course and low twin concordance rates implicate multiple, complex, environmental and epigenetic factors that contribute to MS pathogenesis and most recently, a potential contribution by inducible species of CNS microRNAs (Haghikia et al., 2012; Lopez-Ramirez et al., 2014; Ma et al., 2014; Zhang et al., 2014; Kroesen et al., 2015).

## microRNA (miRNA) up-regulation and target mRNA down-regulation

Human CNS microRNAs (miRNAs) constitute a family of about 2050, 20–23 nucleotide non-coding single stranded RNAs (ssRNAs) that regulate the expression of their target mRNAs post-transcriptionally, and have important roles in development, differentiation, aging, autoimmunity and neurodegeneration (Lukiw et al., 2008; Cui et al., 2010; Guo et al., 2010; O'Connell et al., 2010; Li et al., 2011; Zhang et al., 2014; Zhao et al., 2014).

One major mode of action in the CNS is for inducible, up-regulated miRNAs to decrease their target mRNA levels and hence decrease gene expression (Cui et al., 2010; Guo et al., 2010). Concurrent induction of both NF-kB (p50/p65) and pro-inflammatory miRNAs in stressed human primary brain cells has identified a group of pathogenic miRNAs under NF-kB (p50/p65) transcriptional control and these include miRNA-146a and miRNA-155 (Lukiw, 2012a; Zhang et al., 2014; Zhao et al., 2014). Interestingly, these same miRNAs have been found to be increased in sporadic Alzheimer's disease (AD) tissues which exhibit (i) significant global up-regulation of NF-kB in AD-affected anatomical regions (Lukiw and Bazan, 1998; Lukiw, 2012b); (ii) down-regulation in the expression of innate-immune markers such as the IL-1β receptor-associated kinase 1 (IRAK-1; with a concurrent surge in IRAK-2; Cui et al., 2010); and (iii) a progressive inflammatory degeneration (Latta et al., 2014). Different species of pro-inflammatory miRNAs in different CNS compartments may contribute to similar degenerative pathologies - for example miRNA-146a and miRNA-155 have slightly different effects on inflammatory gene expression in human brain and retina (see below; Lukiw et al., 2012; Ma et al., 2014). It is also important to point out that there appears to be some heterogeneity in miRNA abundance, complexity and related biomarkers amongst different human populations with the same neurological disorder, however recent evidence suggests important common, underlying pathogenic roles for miRNA-146a and miRNA-155 throughout the MS disease process (Meinl and Meister, 2012; Kutty et al., 2013; Harris and Sadiq, 2014; Ma et al., 2014).

## miRNA-146a and inflammatory degeneration

Few CNS-resident miRNAs have gained so much interest as an inducible “*potentially pathogenic miRNA*” implicated in NF-kB-mediated pro-inflammatory signaling and aberrant activation of the innate-immune response as has miRNA-146a, in a surprisingly wide variety of progressive, neurodegenerative CNS disorders. These include AD, MS, and prion-, neurotropic virus- and metal-sulfate-induced neurological dysfunctions (Taganov et al., 2006; Lukiw et al., 2008; Hill et al., 2009; Pogue et al., 2009; Cui et al., 2010; Saba et al., 2012; Kutty et al., 2013; Ma et al., 2014). The 22 nucleotide miRNA-146a (5′-UGAGAACUGAAUUCCAUGGGUU-3′; 59% A + U; NCBI Gene ID: 406938), encoded at human chromosome 5q33.3 is a rapidly induced, pro-inflammatory miRNA with a relatively short half-life of about 1.5–2 h in human CNS cells and tissues (Sethi and Lukiw, 2009). miRNA-146a was originally described as being significantly up-regulated after microbial endotoxin, lipopolysaccharide or cytokine stimulation of myeloid cells and under transcriptional control by NF-κB; shortly thereafter this inducible miRNA-146a was found to be up-regulated by metal sulfate-generated reactive oxygen species, by pro-inflammatory cytokines (such as IL-1β and TNFα) and by Aβ42 peptides in human primary brain cells (Taganov et al., 2006; Lukiw et al., 2008; Hill et al., 2009; Pogue et al., 2009; Cui et al., 2010). Further, miRNA-146a targets the 3′-UTR of mRNAs encoding signaling proteins involved in the innate immune and inflammatory response, including complement factor H (CFH) and IRAK-1, and both compartmentalized CFH and IRAK-1 deficiencies are observed in MS (Taganov et al., 2006; Cui et al., 2010; Ingram et al., 2014; Stürner et al., 2014). Interestingly, significant amounts of miRNA-146a have been found in glial cells responsible for axonal myelination (Li et al., 2011; Alexandrov et al., 2014; Kroesen et al., 2015). Of further interest is that viral infections of brain cells by herpes simplex virus 1 (HSV-1; *Herpesviridae*; Group 1 dsDNA) and other neurotrophic viruses both up-regulate NF-kB and miRNA-146a, and induce pathogenic events that can be quenched using antiviral drugs such as acyclovir (Hill et al., 2009; Lukiw et al., 2010).

## miRNA-155 and neurodegenerative disease

The 23 nucleotide miRNA-155 (5′-UUAAUGCUAAUCGUGAUAGGGGU-3′; 61% A + U; NCBI: AF402776) encoded in humans at chr 21q21.3 is an inducible miRNA under transcriptional control by NF-kB (p50/p65; Lukiw et al., 2012; Lu et al., 2013). miRNA-155, strongly and rapidly up-regulated by inflammatory cytokines, is highly expressed within lymphocytes (both B and T cells) and its role in adaptive and innate immunity has also been strengthened by recent evidence (Lukiw, 2012a,b; Lopez-Ramirez et al., 2014; Seddiki et al., 2014). Specific up-regulation of miRNA-155 is observed in related immunopathologic conditions including MS, Down's syndrome (trisomy 21), rheumatoid arthritis and systemic lupus erythematosus where it affects both T lymphocyte and blood-brain barrier functions (Junker, 2011; Leng et al., 2011; Li et al., 2012; Lopez-Ramirez et al., 2014; Kamphuis et al., 2015). Compared to controls miRNA-155 is the only miRNA commonly increased in MS brain, spinal cord lesions and in peripheral blood mononuclear cells (PBMC; Ma et al., 2014). Interestingly, in experimental autoimmune encephalomyelitis (EAE), a mouse model for MS, miRNA-155 expression has been shown to increase significantly in the spleen, lymph node and brain as EAE progresses, and miRNA-155(–/–) mice exhibit resistance to EAE and significantly less CNS inflammation (Murugaiyan et al., 2011; Thamilarasan et al., 2012). Both miRNA-146a (see above) and/or miRNA-155: (i) target the CD47 3′-UTR, promoting down-regulation of CD47 on brain resident cells, triggering the macrophage-mediated phagocytosis of myelin (Junker et al., 2009) and/or (ii) induce the development of IFN-γ-producing T helper type 1 (Th1) cell subsets and CD4(+) T cells that secrete interleukin (IL)-17 (Th17 cells); these cells are known to induce inflammatory degeneration in MS, psoriasis, autoimmune uveitis and rheumatoid arthritis (Ma et al., 2014; Seddiki et al., 2014; Stürner et al., 2014). Very recently it has been shown that (i) suppressing miRNA-155 expression inhibits the development of Th1 and Th17 cells, resulting in the decrease of disease severity in EAE (Zhang et al., 2014); and (ii) anti-miRNA-155 treatment in EAE significantly inhibits EAE development (Murugaiyan et al., 2011; Ma et al., 2014; Seddiki et al., 2014). As miRNA-155 is encoded on chr21q21.3, the extra gene dosage of chromosome 21 in trisomy 21 patients is associated with (i) increased miRNA-155 levels; (ii) pro-inflammatory signaling and deficits in the innate immune response; and (iii) an increased incidence of autoimmune disease with cognitive disabilities (Lu et al., 2008; Li et al., 2012). The up-regulation of CNS miRNA-155 has also been associated with infection by Bornavirus (*Bornaviridae*; Group V, negative-sense ssRNA) that causes an *enzootic encephalomyelitis*, related to MS with symptoms that include ataxia, ocular disorders, abnormal posture and movement impairment, neurologic disturbances and cognitive/psychiatric disturbances (*http://www.malacards.org/card/borna_disease?search=borna+disease*).

## Anti-miRNA vs. anti-NF-kB strategies

In recent efforts to neutralize NF-kB-triggered, miRNA-mediated pathologies, the use of anti-miRNA and anti-NF-kB strategies have worked surprisingly well both *in vitro* and *in vivo* in animal experimentation. For example anti-miRNA-146a and/or anti-miRNA-155 LNA-protected oligonucleotides administered individually or as combinatorial cocktails exhibited significant efficacy in cytokine-stressed human primary neuronal-glial cell co-cultures and in EAE in reducing aberrant AD- and MS-related pro-inflammatory signaling (Cui et al., 2010; Murugaiyan et al., 2011; Lukiw et al., 2012; Lopez-Ramirez et al., 2014). Very recently miRNA-155 up-regulation that altered junctional organization and permeability of the blood-brain barrier in MS murine models was prevented using inhibition of endogenous miRNA-155 (Lopez-Ramirez et al., 2014; Kamphuis et al., 2015). Equally efficacious appear to be the use of anti-NF-kB remedial strategies; the current number of NF-kB inhibitors now exceeds 900 and the use of combined anti-miRNA and NF-kB inhibitors, and how and when to use them therapeutically, has been recently addressed (Gilmore and Herscovitch, 2006; Lukiw, 2012a,b, 2013; Gibson, 2014). In our view pathogenic miRNA-146a and miRNA-155 up-regulation in several progressive immunodeficiency and/or pro-inflammatory disorders of the CNS indicates that (i) knowledge of the disease mechanism in one neurological disorder may shed some light on a similar disease mechanism in a related CNS disease; (ii) differential anti-miRNA and/or anti-NF-kB therapeutic strategies, perhaps using combinatorial cocktails, should be useful in the clinical management of neurological disorders such as MS; and (iii) multiple NF-kB inhibitors, perhaps combined with multiple anti-miRNA oligonucleotides and current MS pharmacological drugs including dimethyl fumarate and steroids may be tailored to suit each individual MS case in the expanding arena of personalized medicine (Lukiw, 2012a,b; Gotovac et al., 2014; Harris and Sadiq, 2014; Latta et al., 2014).

## Concluding remarks

Our understanding of NF-kB-regulated miRNAs and their abundance and complexity are revolutionizing our perceptions and ideas on gene expression in CNS aging and disease. *It is our opinion that (i) progressive inflammatory neurodegeneration in MS involves NF-kB-regulated miRNA-146a and miRNA-155 and perhaps other pathogenic miRNAs which are inducible; (ii) targeting of inflammation-relevant gene expression by miRNA-146a and/or miRNA-155 suggest pathogenic pathways of MS may be in common with other kinds of human CNS degenerations; (iii) altered miRNA expression patterns in MS may be useful both diagnostically and in the design of novel therapeutic approaches; and (iv) anti-miRNA-146a, anti-miRNA-155, anti-NF-kB and perhaps anti-viral drugs, either alone or in combination with currently prescribed MS anti-inflammatory drugs, should open new avenues for future MS research and therapeutic strategies useful in the clinical management of MS and other CNS disorders with a progressive inflammatory and degenerative component*.

### Conflict of interest statement

The authors declare that the research was conducted in the absence of any commercial or financial relationships that could be construed as a potential conflict of interest.
